# Supplementary material for: Multi‐dataset identification of innovative feature genes and molecular mechanisms in keratoconus
Source: J Cell Mol Med. 2024 Sep 19;28(18):e70079. doi: 10.1111/jcmm.70079 (PMC11412914; doi:10.1111/jcmm.70079)
Supplement: Supplementary file 2 — Material S2. [file JCMM-28-e70079-s002.zip › 7.GSEA analysis/2.ATOH7/KEGG_TERPENOID_BACKBONE_BIOSYNTHESIS.html]

Details for gene set KEGG\_TERPENOID\_BACKBONE\_BIOSYNTHESIS[GSEA]

|  || Dataset | ATOH7.group.cls#h\_versus\_l |
| Phenotype | group.cls#h\_versus\_l |
| Upregulated in class | l |
| GeneSet | KEGG\_TERPENOID\_BACKBONE\_BIOSYNTHESIS |
| Enrichment Score (ES) | -0.31844226 |
| Normalized Enrichment Score (NES) | -0.8327168 |
| Nominal p-value | 0.634 |
| FDR q-value | 1.0 |
| FWER p-Value | 1.0 |
Table: GSEA Results Summary

  

Fig 1: Enrichment plot: KEGG\_TERPENOID\_BACKBONE\_BIOSYNTHESIS      
 Profile of the Running ES Score & Positions of GeneSet Members on the Rank Ordered List

  

| SYMBOL | TITLE | RANK IN GENE LIST | RANK METRIC SCORE | RUNNING ES | CORE ENRICHMENT || 1 | IDI2 | na | 5439 | 0.187 | -0.0178 | No |
| 2 | DHDDS | na | 8059 | 0.118 | -0.0059 | No |
| 3 | GGPS1 | na | 9925 | 0.083 | 0.0017 | No |
| 4 | PDSS2 | na | 12880 | 0.042 | -0.0524 | No |
| 5 | ACAT1 | na | 15830 | 0.014 | -0.1269 | No |
| 6 | PMVK | na | 20605 | -0.028 | -0.2438 | No |
| 7 | HMGCS2 | na | 23196 | -0.057 | -0.2758 | Yes |
| 8 | ACAT2 | na | 23817 | -0.067 | -0.2440 | Yes |
| 9 | HMGCR | na | 23968 | -0.069 | -0.1970 | Yes |
| 10 | HMGCS1 | na | 24316 | -0.074 | -0.1520 | Yes |
| 11 | IDI1 | na | 24690 | -0.080 | -0.1030 | Yes |
| 12 | FDPS | na | 24862 | -0.083 | -0.0462 | Yes |
| 13 | PDSS1 | na | 25728 | -0.099 | 0.0021 | Yes |
| 14 | MVK | na | 27087 | -0.128 | 0.0577 | Yes |
| 15 | MVD | na | 30224 | -0.219 | 0.1297 | Yes |
Table: GSEA details [plain text format]

  

Fig 2: KEGG\_TERPENOID\_BACKBONE\_BIOSYNTHESIS      
 Blue-Pink O' Gram in the Space of the Analyzed GeneSet

  

Fig 3: KEGG\_TERPENOID\_BACKBONE\_BIOSYNTHESIS: Random ES distribution      
 Gene set null distribution of ES for **KEGG\_TERPENOID\_BACKBONE\_BIOSYNTHESIS**
